# Minichromosome Maintenance Proteins: From DNA Replication to the DNA Damage Response

**DOI:** 10.3390/cells14010012

**Published:** 2024-12-26

**Authors:** Agnes Malysa, Xiaohong Mary Zhang, Gerold Bepler

**Affiliations:** Karmanos Cancer Institute, Department of Oncology, School of Medicine, Wayne State University, 4100 John R Street, Detroit, MI 48201, USA; malysaa@karmanos.org (A.M.); ga5554@wayne.edu (X.M.Z.)

**Keywords:** minichromosome maintenance (MCM), DNA replication, origin recognition complex (ORC), pre-replication complex (pre-RC), cell cycle checkpoint, replication stress, DNA damage response (DDR)

## Abstract

The DNA replication machinery is highly conserved from bacteria to eukaryotic cells. Faithful DNA replication is vital for cells to transmit accurate genetic information to the next generation. However, both internal and external DNA damages threaten the intricate DNA replication process, leading to the activation of the DNA damage response (DDR) system. Dysfunctional DNA replication and DDR are a source of genomic instability, causing heritable mutations that drive cancer evolutions. The family of minichromosome maintenance (MCM) proteins plays an important role not only in DNA replication but also in DDR. Here, we will review the current strides of MCM proteins in these integrated processes as well as the acetylation/deacetylation of MCM proteins and the value of MCMs as biomarkers in cancer.

## 1. Introduction

In 1984, Chinese-American molecular geneticist and structural biologist Dr. Bik-Kwoon Yeung Tye at Cornell University first discovered the minichromosome maintenance (MCM) genes in yeast [1]. Her lab conducted a genetic screen for budding yeast mutants that were defective in maintaining a minichromosome that contained a cloned replication origin and centromere [1]. The genes that could rescue these mutants were named minichromosome maintenance (MCM) genes: examples include *MCM2*, *MCM3*, *MCM5 and MCM10* [2].

**Figure 1:**
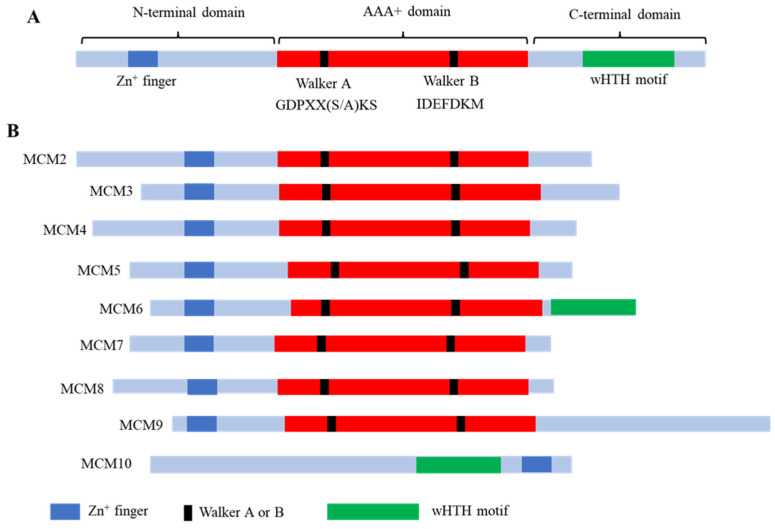
The domain structures of MCM family proteins. (**A**) Three domains of MCM family proteins. (**B**) The domain structures of MCM family proteins: MCM2-10. wHTH stands for the winged helix-turn-helix motif. Please see the text for more details.

The MCM proteins belong to a protein family called the AAA+ (ATPases Associated with diverse cellular Activities) protein superfamily [3,4,5]. As its name indicates, all the members within this superfamily have conserved ATPase domains and participate in ATP-dependent cellular activities like DNA replication [2,6,7]. In general, the primary structure of MCM proteins contains three domains: the N-terminal domain, the middle AAA+ domain or the MCM box, and the C-terminal domain (Figure 1). The N-terminal domain contains the zinc-binding motif, which contributes to complex assembly and ATPase activity. The MCM box includes two ATPase consensus motifs: the Walker A motif, containing the MCM-specific consensus GDPxx(S/A)KS, and the Walker B motif, containing the sequence IDEFDKM, which is conserved in all MCM proteins and defines the MCM family [8,9]. The C-terminal domain contains a winged helix domain, which binds to DNA. The nuclear localization sequences (NLSs) only exist in MCM2 and MCM3. To date, nine MCM subfamilies have been identified: MCM2, MCM3, MCM4, MCM5, MCM6, MCM7, MCM8, MCM9, and MCM10, where MCM10 does not share sequence homology and has a distinct structure so we will only focus on its importance to DNA replication with MCM2–7 [8,9]. Although MCM1 was identified in the same genetic screen, it functions distinctly from the rest of the MCM proteins: MCM1 is a transcription factor [10] that regulates the transcription of DNA replication genes, including MCMs [11]. Thus, MCM1 is not discussed in this review. 

Structurally, compared to MCM2–7, MCM9 has a long C-terminal domain. The layout of its C-terminal domain mimics yeast Srs2 helicase, which has been shown to regulate DNA replication and repair proteins—for instance, by proliferating cell nuclear antigen (PCNA) and Rad51 [12]. This structural disparity suggests the differential roles among the MCM2–7 complex, MCM10, and the MCM8/9 complex in DNA replication. 

It is well known that the MCM2–7 complex serves as replicative helicases to unwind the DNA double helix at the origins of replication by breaking down the hydrogen bonds between two DNA strands. Subsequently, the DNA duplex can be unzipped to form replication forks, allowing replication to occur on each single strand. To make sure that the faithful duplication of the genome only happens once per cell cycle, the DNA replication process is highly regulated [5,13,14]. Any condition that disrupts or compromises this process is referred to as replication stress, which is a major cause of genome instability, a hallmark of cancer [9,15]. Replication stress then activates the DNA damage response (DDR), an intricate surveillance system to protect stalled forks and promote replication completion [16]. As part of DDR, DNA repair pathways, such as homologous recombination (HR), restore replication upon DNA breaks that occur at stalled forks. Given the intimate relationship between DNA replication and DDR, the role of MCMs could be extended to DDR [9,15]. 

When interrogating the relationship between MCMs and chromatin, the results revealed, surprisingly, that a large fraction of MCMs is not associated with replication origins [2,3]. This abundance of MCMs has been denoted in the literature as the “MCM paradox” [17,18]. Further studies have shown that when MCM pools are reduced, there is no impact on DNA replication [19,20,21], which begs the following question: What is the purpose of these excess MCMs? While some researchers have observed these excess MCMs in the form of dormant replication origins as backups for active origins, the answer is still largely unknown. Others have postulated the purpose of these excess MCM pools to be transcriptional regulation [22,23,24,25,26], chromatin remodeling [27,28], or DNA damage checkpoint response [15,19,20,21,22,23,24,25,26,27,28,29,30,31,32]. Overall, current research suggests that these excess MCM pools may serve as a link between DNA replication and DDR.

Here, we will summarize recent advances in research concerning MCMs in DNA replication and DDR as well as in cancer. As phosphorylation is a dominant post-translation modification (PTM) in regulating the MCM complex, other PTMs attract less attention. In this article, we will also briefly discuss the new research advances concerning acetylation/deacetylation in MCMs. 

## 2. MCMs and the Pre-Replication Complex (Pre-RC) in DNA Replication 

### 2.1. The MCM-Associated Proteins in DNA Replication

Origin recognition complex (ORC) proteins were first discovered from budding yeast [33,34]. There are six members in this family, including Orc1 through Orc6, which are bound to DNA replication origins throughout the cell cycle. They function as ATP-dependent enzymes binding to specific DNA sequences at the origins of replication to initiate the process of DNA replication in eukaryotic cells. They also serve as scaffold proteins to recruit key factors to form pre-replication complexes (pre-RCs) [35]. Orc1–6 have conserved AAA+ domains, including Walker A and Walker B ATP-binding motifs, characteristic of ATP-dependent clamp-loading proteins, which allow ring-shaped protein complexes to encircle duplex DNA [35]. 

Cell division cycle 6 (Cdc6) is an important regulator of DNA replication in eukaryotic cells [33]. It is essential for the assembly of pre-RC at the origins of replication during the G1 phase of the cell cycle. Unlike nearly all other licensing factors, which are AAA+ ATPases, the cell division cycle 10 (Cdc10)-dependent transcript 1 protein (Cdt1) is not an enzyme [36]. The Cdt1 protein sequence bears little resemblance to other proteins of known molecular activity. It is essential for origin licensing in all eukaryotes tested. 

### 2.2. MCM2–7, MCM8/9 and MCM10 Function in DNA Replication

For DNA replication to occur, studies observed that at the end of the M phase and the beginning of the G1 phase, Cdc6 and Orc1–6 scout for areas of DNA that need to be replicated. Once they find these replication origins, Orc1–6 forms a scaffold for the MCM2–7 double hexamer to be loaded with the help of replication licensing factor Cdt1 [3,5]. This complex is referred to as the pre-replication complex (pre-RC), where loading the MCM2–7 double hexamer and formation of the pre-RC signifies a phenomenon called origin DNA licensing, which is a process that ensures only one round of DNA replication occurs during the cell cycle [37,38]. 

Once the MCM2–7 double hexamer is loaded, ORC disengages, and MCM10, with the help of cyclin-dependent kinase/dumbbell former 4 (DBF4)-dependent kinase (CDK/DDK), aids in loading cell division cycle protein 45 (Cdc45) to form the Cdc45-MCM-GINS (CMG) helicase [39]. GINS represents “Goichi-Nisan”, which is an acronym derived from the Japanese numbers “5-1-2-3”, referring to the four protein subunits that make up the GINS complex: Sld5 (synthetic lethal with Dpb11), Psf1 (partners of Sld-five 1), Psf2, and Psf3. Dpb11 stands for DNA polymerase B (II). CDK/DDK not only are involved in loading replication factors but also play a role in activating the CMG helicase through the phosphorylation of MCM2, which is the event that turns on the CMG helicase and initiates origin firing to start DNA replication [40]. 

Notably, the MCM2–7 proteins form a heterohexamer, which is a critical component of the human replicative Cdc45-MCM-GINS (CMG) helicase, as it controls helicase activity [17,20,21]. The MCM2–7 hexamer is formed in the following manner: MCM subunits 4/6/7 bind tightly to each other to form the MCM core, and MCM2 and the MCM3/5 dimer bind to this MCM4/6/7 core [3]. It was observed that the MCM4/6/7 core was critical for regulating helicase function because mutating MCM 4/6/7 disturbed helicase activity [3]. Additionally, when MCM7 was mutated, there was a decline in ATPase and helicase abilities; however, when MCM4 was mutated, this downregulation in MCM4 led to an increase in helicase activity which highlighted MCM7’s regulatory effects and MCM4’s inhibitory effect on the CMG helicase [37]. 

MCM8 and MCM9 play a peripheral role in DNA replication where they interact with each other to form a complex (MCM8/9) which is responsible for regulating the integrity of the replication fork. The MCM8/9 complex does not interact with the MCM2–7 double hexamer, but it does interact with the DNA replication machinery [12,41,42]. MCM8/9 has been observed to be responsible for regulating fork progression, restart and reversal. For fork progression and restart, cells deficient in MCM2 would use MCM8/9 to maintain DNA synthesis, presumably through MCM8/9’s helicase abilities [12,41,42]. The MCM8/9 complex is not known for directly affecting fork reversal, but it interacts with DNA repair proteins (like Rad51 and Mre11), which are involved in fork reversal [12].

MCM10 has been implicated in DNA initiation and elongation. With respect to DNA initiation, early studies demonstrated that MCM10 interacts with the MCM2–7 double hexamer and mediates the recruitment of Cdc45 to activate CMG helicase [43]. This recruitment occurs independently of CDK and DDK [44]. For DNA elongation, MCM10 has been observed at the replication origins where many studies have shown that MCM10 interacts with DNA polymerase α [39]; through this association, it was surmised that MCM10 links helicase’s unwinding of DNA and DNA synthesis [45]. Newer studies have further clarified the role of MCM10 in DNA replication. It was determined that MCM10’s C-terminal domain binds to the MCM2–7 double hexamer on chromatin, an interaction critical for promoting splitting of the double hexamer MCM2–7 for bidirectional DNA unwinding or placing MCM10 at the front of the replication fork, where it removes replication protein A (RPA) and aids in reannealing DNA strands [46].

## 3. MCMs and DDR

### 3.1. MCM2–7 and DDR

Multiple lines of evidence indicate that MCMs play critical roles in DDR. Research in yeast has demonstrated that MCMs physically interact with DNA repair proteins through their associations with the replication machinery [3,9,47,48,49]. For example, RPA has been shown to associate with MCM7 at early origins of replication, while Rad53, along with CDKs and DDKs, regulates these coordinated processes [50,51]. In humans, the functional equivalent of Rad53 is checkpoint kinase 2 (Chk2), a key component of the ATM/ATR signaling pathway [52]. As it will be explored below, MCM2–7 proteins are closely linked to the ATM/ATR pathway among other DNA repair pathways, further underscoring their involvement in DDR.

#### 3.1.1. MCM2 and MCM3 in the ATM/ATR Pathway

Cortez et al. demonstrated that MCM2 and MCM3 are phosphorylated by ATM and ATR, respectively, in response to various DNA-damaging agents [30,53,54]. This phosphorylation of MCM2 by ATM/ATR has been further corroborated by multiple research groups [31,49,55,56]. Ibarra et al. reported that knocking down MCM3 in the presence of DNA-damaging agents led to defects in S phase progression, heightened sensitivity to replicative stress, and activation of checkpoint kinase 1 (Chk1) and checkpoint kinase 2 (Chk2) [19]. Similarly, Li et al. discovered an interaction between MCM3 and Cyclin E/Cdk2, showing that overexpression of MCM3 resulted in S phase arrest and upregulation of Chk1 [54]. Additionally, mutations in MCM2 were shown to increase mutation rates upon exposure to genotoxic drugs [54], while DDK-mediated phosphorylation of MCM2 was found to stabilize replication forks during replicative stress [57].

Further studies have elucidated the role of MCM2 within the ATM/ATR pathway. Tsai et al. examined the yeast mutant *mcm2DENQ*, which carries a mutation in the Walker B ATPase motif and observed defective Rad53 (human Chk2) phosphorylation only after activating a DNA replication checkpoint [58]. This finding suggests that the MCM2 mutation impairs Rad53 phosphorylation within the checkpoint pathway [58]. Expanding on this, Vijayraghavan et al. found that *mcm2DENQ* mutants treated with DNA-damaging agents exhibited elevated levels of spontaneous DNA damage during the G2/M phase, with DNA damage foci occurring predominantly in this phase rather than during S phase [59].

Additional research has revealed broader roles for MCM2 and MCM3 in the ATM/ATR repair pathway. Drissi et al. showed that knocking down MCM2 and MCM3 in the presence of DNA-damaging agents led to decreased activation of Chk1 and Chk2, as well as impairments in homologous recombination (HR) and non-homologous end joining (NHEJ) repair [31]. These findings collectively highlight the integral roles of MCM2 and MCM3 in the ATM/ATR pathway and their broader contributions to maintaining genomic stability under conditions of replicative stress and DNA damage [31].

#### 3.1.2. MCM4 and MCM6 in the ATM/ATR Pathway

MCM4 has been identified as a key player in the DNA damage response (DDR). Chk1, a checkpoint protein that signals DNA repair, phosphorylates MCM4 to prevent DNA synthesis, highlighting the involvement of MCMs in the DDR [60]. Additionally, Forsburg et al. demonstrated that MCM4 interacts with Cds1, a checkpoint kinase homologous to human Rad53, and Rhp51, a yeast homologous repair protein, suggesting that MCM4 is actively involved in homologous recombination repair (HRR) [15].

Further investigations by Sheu et al. revealed that deleting proximal regions of the serine/threonine-rich domain (NSD) in the N-terminus of yeast MCM4 caused late origin firing in response to DNA-damaging agents, despite an otherwise robust ATM/ATR-mediated DDR [61]. This finding led to the conclusion that MCM4’s NSD region regulates checkpoint signaling to prevent late origin firing. Han et al. expanded on this by showing that Chk1 interacts with all components of the MCM2–7 hexamer [53]. While the interaction between Chk1 and MCMs decreases during DNA damage, MCM proteins continue to facilitate Chk1 recruitment to chromatin, further elucidating their role in the ATM/ATR pathway.

Additionally, the role of MCM4 in checkpoint activation was explored by Wallace et al., who crossed mutant MCM4 mice with ATM-null mice. These mice exhibited increased tumor susceptibility, shorter tumor latency, and accumulated double-strand breaks (DSBs) due to collapsed or stalled replication forks [62]. The absence of compensatory dormant origin activation and DDR signaling exacerbated the accumulation of unrepaired DNA [62]. Furthermore, Forsburg et al., a research group that has been instrumental in studying MCMs through yeast models, demonstrated that knocking down MCM4 and MCM7 in human cells resulted in chromosomal abnormalities and cell death, emphasizing the essential role of these proteins in genome stability [3,9,15]. Similarly, Forsburg et al. revealed that mutant MCM yeast strains treated with DNA-damaging agents exhibited irreversible DNA damage [3,9,15]. Among these, mutants sustained the most significant damage, highlighting the sensitivity of specific MCM components to genotoxic stress.

MCM6 also plays a significant role within the ATR pathway. Komata et al. identified an interaction between MCM6 and mannose receptor C-type 1 (Mrc1), a checkpoint protein involved in ATR/Chk1 signaling [63]. Mutated MCM6, when fused with wild-type Mrc1, restored checkpoint function in yeast treated with DNA-damaging agents. This finding indicates that MCM6 acts as a sensor for checkpoint activation [63]. Other studies confirmed that MCM6 is a direct target of ATR during DNA damage, reinforcing its role in DDR [64,65].

Collectively, these findings highlight the importance of MCM2–7 proteins in the DDR. Notably, Yun et al. observed that treating human colorectal adenocarcinoma cells with DNA-damaging agents downregulated MCM4 expression, whereas other MCM proteins remained unaffected [66]. Prolonged treatment, however, led to the decline of all MCM2–7 protein levels. Based on these results, the researchers concluded that MCM4 is the first to respond to DNA damage signals and likely coordinates its response with p53 and Chk2 [66].These studies demonstrate the intricate roles of MCM4 and MCM6 in maintaining genome stability through their involvement in the ATM/ATR pathway and DDR. 

#### 3.1.3. MCM7 in the ATM/ATR Pathway

Cortez et al. identified a physical interaction between MCM7 and ATR-interacting protein (ATRIP), a key partner of ATR that initiates DDR [30,67]. Their findings demonstrated that reducing MCM7 levels disrupts the intra-S phase checkpoint. Building on this, Tsao et al. uncovered a novel interaction between human Rad17, a checkpoint protein phosphorylated by ATR, and MCM7 [68]. The depletion of either Rad17 or MCM7 was shown to inhibit Chk1 phosphorylation. Furthermore, they observed that the loss of MCM7 disrupted the formation of ATR nuclear foci in response to DNA damage and caused defects in the intra-S phase checkpoint.

Wei et al. further confirmed the role of MCM7 in S phase checkpoint regulation. They demonstrated that cyclin E/Cdk2 and cyclin B/Cdk1 phosphorylate MCM7, and that MCM7 overexpression delays S phase entry by activating Chk1 in a p53-dependent manner [69]. This reinforces MCM7’s critical role in orchestrating DDR and maintaining cell cycle integrity. These findings collectively underscore the pivotal function of MCM7 in the ATM/ATR pathway, particularly in regulating the intra-S phase checkpoint and ensuring proper DDR signaling.

#### 3.1.4. MCMs and the Fanconi Anemia Pathway and Homologous Recombination Repair

Emerging evidence highlights the role of MCM2–7 proteins in DNA repair pathways other than the ATM/ATR pathway. Drissi et al. demonstrated that MCM2 interacts with ASF1 (Anti-Silencing Function 1), a histone chaperone and chromatin remodeling factor that also antagonizes BRCA1-dependent DNA end resection and stimulates non-homologous end joining (NHEJ) [32]. This interaction was notably enhanced following treatment with DNA-damaging agents, with the colocalization of MCM2 and ASF1 observed at DNA damage foci. These findings provide valuable insights into the role of MCMs in chromatin-level responses to DNA damage.

Yu et al. identified a novel interaction between MCM2/3/5/6 and mediator of DNA damage checkpoint protein 1 (MDC1), a key DDR protein recruited to DNA break sites in homologous recombination repair (HRR). Knocking down MCM2/3/5/6 resulted in reduced MDC1 expression and impaired DNA damage foci formation, further emphasizing the role of MCM proteins in facilitating DDR processes [70].

The connection between MCMs and the Fanconi anemia (FA) pathway—a critical DNA repair mechanism associated with a predisposed childhood cancer syndrome—has also been examined [71]. FA proteins are essential for maintaining genomic stability and interact with ATR to initiate the DNA damage response [72]. In this pathway, the FA core complex mono-ubiquitinates Fanconi anemia complementation group D2 (FANCD2) and Fanconi anemia complementation group I (FANCI) to promote DNA repair. Lossaint et al. revealed that FANCD2, a key FA protein involved in cell cycle regulation and DNA repair, interacts with MCM2/3/5/7 [73]. Furthermore, ATR was shown to be necessary for mediating the interaction between FANCD2 and MCM proteins, underscoring the role of MCMs in the FA pathway.

These findings collectively highlight the multifaceted roles of MCMs in DNA repair, particularly their contributions to the Fanconi anemia pathway and broader chromatin-associated repair mechanisms.

The involvement of MCMs in HRR has been highlighted in several studies. Shukla et al. demonstrated that MCM2 and MCM3 interact with key HR proteins Rad51 and Rad52 [74]. Building on this, Cabello-Lobato et al. found that Rad51 and Rad52 bind to MCM4, regardless of whether DNA damage is present [74]. Their research further revealed that the MCM4/Rad51 complex facilitates replication fork progression under conditions of DNA damage, independent of Rad51’s recombination activity.

Additionally, Chen et al. observed that MCM2/3/5/6 interact with 53BP1, a protein that localizes to double-strand break (DSB) sites and activates p53 in HRR [72]. Knocking down MCM2 or MCM6 led to a reduction in 53BP1 foci formation and its association with chromatin, further emphasizing the critical role of MCMs in DNA repair [72].

To expand on this understanding, Stead et al. demonstrated that ATR phosphorylates MCM2 to initiate DNA repair, underscoring the central role of the MCM2–7 complex in maintaining genomic stability through DNA repair mechanisms [57,75]. These findings collectively highlight the multifaceted roles of MCMs in facilitating HRR and responding to DNA damage.

### 3.2. MCM8/9 and DDR

While the above studies have shown that MCM8/9 may act as a compensatory helicase when the MCM2–7 hexamer is dysfunctional, the MCM8/9 complex has also been shown to have an association with DNA repair [76]. Lutzmann et al. discovered that MCM8/9 forms a stable complex and when MCM8 or MCM9 expression was depleted, mouse models demonstrated substantial genomic instability and growth defects [77]. Lutzmann et al. also discovered that MCM8 depletion blocked HR-mediated DSB repair, which led the research group to conclude that MCM8/9 regulates HR [77] Interestingly, Nishimura et al. discovered that null MCM8 or MCM9 chicken cells were sensitive to various DSB damaging agents, which led them to conclude that MCM8 and MCM9 were resistant to DNA damage [78]. They also discovered that the MCM8/9 complex possibly works as a helicase in HR repair, downstream of the Fanconi anemia and BRCA2/Rad51 repair pathways. Further investigating MCM8/9’s role in HR repair, Lee et al. discovered that MCM8 and MCM9 interact with the MRN complex (consisting of Mre11, Rad50 and Nbs1 in yeast) and help recruit MRE11 to DSB sites, leading the researchers to conclude that the MCM8/9 complex was vital for HR repair [79]. Hustedl et al. built upon this discovery and observed that homologous recombination factor with OB (oligosaccharide-binding domain)-fold (HROB), a key protein involved in HR repair, recruits MCM8/9 to the DNA damage sites [80] In addition, Huang et al. discovered another protein within the HR repair pathway, MCM8IP (MCM8-interacting protein), which stimulates MCM8/9’s helicase activity and promotes DNA replication for DNA repair [81]. Other recent studies have continued to examine the function and structure of MCM8/9 in the HR repair pathway [77,78,82]. In addition to HR repair, the MCM8/9 complex has also been implicated in the mismatch repair pathway (MMR): Traver et al. discovered that MCM9 interacts with MSH2 (mutator S homolog 2), MSH3 (mutator S homolog 3), MLH1 (mutator L homolog 1) and PSM1 (postmeiotic segregation increased 1)—key proteins in MMR—and that these MMR proteins, together with MCM8 and MCM9, function as a helicase with this repair pathway [83]. In addition, Liu et al. discovered that HORMA domain-containing protein 1 (HORMAD1) interacts with MCM8/9 and prevents MCM8/9 nuclear localization when overexpressed [84]. HORMA is an acronym for the initial letters of three proteins that contain the HORMA domain: Hop1, Rev7, and Mad2. They also discovered that HORMAD1’s interaction with MCM8/9 compromises MMR: HORMAD1 knockout (KO) cells were observed to have a higher sensitivity to 6-thioguanine (6-TG), a DNA damaging agent whose resistance is associated with MMR deficiency. All these findings suggest that MCM8/9 has a potential role in DNA repair.

#### Auxiliary MCM8/MCM9 Helicases in DDR

The auxiliary helicases MCM8 and MCM9 are critical regulators of HRR. Unlike the canonical MCM2–7 helicase complex, MCM8/9 operates specifically during HRR to facilitate the resolution of double-strand breaks (DSBs) [79]. MCM8/9 forms a stable heterodimer that localizes to damaged DNA sites and interacts with key HRR proteins, including RAD51 and BRCA1/2, to promote strand invasion and repair synthesis [81]. Mutations or the depletion of MCM8/9 lead to defective DSB repair, chromosomal aberrations, and increased sensitivity to DNA-damaging agents, underscoring their crucial role in HR repair [85].

MCM8/9 also plays a pivotal role in regulating DNA end resection, the process that generates single-stranded DNA (ssDNA) necessary for RAD51 filament formation and strand exchange during HRR^12^. Specifically, MCM8/9 collaborates with resection machinery, including exonucleases like exonuclease 1 (EXO1) and DNA replication helicase/nuclease 2 (DNA2), to promote long-range resection beyond the initial processing step mediated by CtIP (carboxy-terminal binding protein interacting protein) and the MRN (Mre11, Rad50, and Nbs1) complex [85]. This activity ensures an efficient transition to the later stages of HRR. Notably, MCM8/9’s regulation of end resection is tightly controlled to prevent excessive ssDNA generation, which could lead to genomic instability [86]. By coordinating end resection and repair activities, MCM8/9 ensures the accurate repair of DSBs and preserves genomic integrity [72]. 

### 3.3. MCM10 and DDR

The association between MCM10 and DDR is not quite as clear. There are very few studies that have highlighted the significance of this relationship. From what is currently known in the literature, MCM10 has been shown to affect cell cycle checkpoint activation and DSB repair. With respect to checkpoint activation, Park et al. demonstrated that the knockdown of MCM10 activates Chk1 and Chk2 to inhibit Cdc25, a cyclin that regulates the G2/M phase [87]. This research group also observed that Chk1 regulates the G2 arrest in MCM10-depleted cells and that prolonging MCM10 knockdown causes DSB and eventually cell death. Investigating MCM10’s effect on DSB repair, Chattopadhyay et al. discovered that MCM10 and DNA polymerase α catalytic subunit (p180) interacts with each other such that MCM10 knockdown causes a destabilization of p180 and leads to DSB [39]. Sharma et al. examined how different types of radiation affect MCM10 and its response to DNA repair. They discovered that UV radiation would downregulate MCM10 expression and mediate a UV-directed DSB repair [88]. Kang et al. observed that MCM10 binds to DNA damaged repair proteins BRCA2 (Breast Cancer gene 2) and PALB2 (partner and localizer of BRCA2)—key proteins in HR repair—and found that, at the time of DNA damage, the association between BRCA2 and MCM10 prevented replication fork progression, suggesting that MCM10 has a potential role in DNA replication [89].

## 4. Intersection of MCMs, R-Loop Resolution and DDR

MCMs are central to DNA replication and genome stability, with emerging evidence highlighting their critical role in resolving R-loops—three-stranded structures formed by an RNA:DNA hybrid and a displaced single-stranded DNA [90,91]. These R-loops can arise during transcription and, if unresolved, contribute to replication stress, DNA damage, and genomic instability [90]. The MCM2–7 complex has been implicated in facilitating R-loop removal by cooperating with factors such as RNase H enzymes and DNA repair pathways [92,93]. By preventing the accumulation of R-loops, MCMs help maintain a delicate balance between transcription and replication processes, thereby protecting cells from transcription-associated genome instability [92,93]. This function highlights the significance of MCMs in safeguarding genomic integrity beyond their well-established role in DNA replication initiation.

In addition to their role in R-loop resolution, MCMs are pivotal components of DDR, where they contribute to stabilizing stalled replication forks and facilitating repair pathways such as HRR [30,67,94]. By maintaining fork stability and activating repair mechanisms, MCMs play a dual role in mitigating genome instability and preventing DNA damage accumulation. This coordination between replication and transcription processes positions MCMs as critical buffers against genomic instability, a hallmark of cancer and other disorders characterized by DNA damage [30,67,94]. These multifaceted roles emphasize the importance of MCMs in protecting the genome and highlight their potential as therapeutic targets in diseases associated with genome instability.

## 5. Acetylation/Deacetylation of MCMs

Recent studies have highlighted the critical roles of MCMs in integrating DNA replication, DDR, and transcriptional regulation [91]. Advances in understanding their molecular functions reveal that MCM proteins not only drive replication fork progression but also contribute to R-loop resolution and replication-transcription conflict management, ensuring genome stability [91]. These findings have expanded to include post-translational modifications, particularly acetylation and deacetylation, which modulate MCM protein activity, stability, and interactions. For example, MCM10, essential for replication initiation, is acetylated and can be deacetylated by the sirtuin family deacetylase SIRT1, which promotes replication fork initiation and stabilizes MCMs at replication origins [95]. Similarly, MCM3AP (MCM3 associated protein), an acetyltransferase specific to MCM3, acetylates its target to regulate DNA replication [96]. The overexpression of MCM3AP inhibits replication, underscoring the importance of acetylation in controlling replication fidelity and its potential links to genomic instability in disease contexts [96].

The reversible acetylation of MCMs represents a finely tuned regulatory mechanism that integrates DNA replication with cellular stress responses. Acetylation enhances the stability and DNA-binding ability of MCMs, while deacetylation restores their dynamic interactions necessary for replication progression and damage repair [97]. These modifications also provide a link to cancer biology, as aberrant acetylation patterns of MCMs have been associated with tumorigenesis [97]. For instance, the dysregulation of MCM3AP activity and its acetylation motifs can contribute to uncontrolled DNA replication or genomic instability, hallmarks of cancer cells. Furthermore, SIRT1-mediated deacetylation of MCM10 may be disrupted in cancer, potentially leading to replication stress and increased susceptibility to DNA damage. These connections underscore the need to explore MCM acetylation pathways as potential therapeutic targets.

Given their central role in replication, DDR, and transcription, MCMs are emerging as valuable biomarkers for cancer diagnosis, prognosis, and treatment stratification. Elevated levels of MCMs have been observed in various cancers, correlating with increased proliferative capacity and poor prognosis [98]. For example, MCM2 and MCM7 expression levels are often upregulated in aggressive tumors and may serve as indicators of tumor grade and replication stress [98,99]. Moreover, post-translational modifications such as acetylation could further refine their utility as biomarkers by providing additional insights into the functional state of MCMs in tumor cells. Advances in proteomics and acetylation mapping techniques could enable the development of targeted therapies that exploit MCM modifications to selectively disrupt replication or DDR processes in cancer cells, offering a promising avenue for precision oncology.

## 6. Targeting MCMs in Cancer Therapeutics

MCMs are coming into view as potential targets for cancer therapy due to their critical roles in DNA replication and repair, processes that are often dysregulated in cancer. The MCM2–7 complex, is frequently overexpressed in various cancers and correlates with poor prognosis [99]. This overexpression contributes to the replication stress and genomic instability characteristic of cancer cells, making MCMs attractive therapeutic targets [100]. Inhibitors targeting the helicase activity of the MCM complex or its interactions with other replication factors have shown promise in preclinical studies by selectively inducing DNA damage and apoptosis in cancer cells without affecting normal cells.

Efforts to target MCMs have also focused on their auxiliary roles in DNA repair pathways, such as HRR. As previously mentioned, MCM8 and MCM9 are involved in HRR and are essential for maintaining genomic stability. Inhibiting these proteins has been proposed as a strategy to enhance the efficacy of DNA-damaging chemotherapies or radiotherapy by impairing the repair of therapy-induced DNA damage. Small molecule inhibitors and RNA interference approaches targeting MCMs are currently under investigation, with early studies indicating that MCM inhibition sensitizes cancer cells to replication stress and DNA damage [101,102]. These findings suggest that MCMs are not only targetable but may also improve the therapeutic window of existing cancer treatments.

## 7. Open-Ended Questions for the MCMs and DDR Relationship and How to Address These Knowledge Gaps

Despite significant advancements in understanding the roles of MCMs in DNA replication and genome stability, key knowledge gaps remain in elucidating their precise relationship with DDR. One notable gap concerns how the MCM complex senses and responds to different types of DNA damage. While the MCM2–7 helicase is known to stabilize stalled replication forks and interact with DDR factors such as ATR and CHK1, the specific molecular mechanisms by which MCMs coordinate these activities are poorly understood [2,103]. Additionally, it is unclear whether all MCMs have unique or redundant functions in DDR, as studies have predominantly focused on MCM2–7, leaving the roles of MCM8, MCM9 and MCM10 less explored. A better understanding of how MCM proteins discriminate among different types of DNA damage could provide valuable insights into their broader roles in genome maintenance.

Another significant knowledge gap lies in the crosstalk between MCMs and transcription-associated DNA damage. R-loops, which arise during transcription and are a major source of replication stress, are resolved in part by MCM proteins. However, the exact mechanisms by which MCMs coordinate R-loop resolution with DDR pathways remain elusive. For example, it is unclear how MCM helicase activity is regulated in response to R-loop accumulation or how it interfaces with RNA processing enzymes and repair machinery. Additionally, the potential roles of post-translational modifications of MCMs, such as phosphorylation or ubiquitination, in modulating their DDR functions have not been systematically investigated. Understanding these processes is critical, as defects in R-loop resolution and DDR coordination are linked to diseases such as cancer and neurodegenerative disorders.

Addressing these knowledge gaps will require a combination of advanced experimental approaches. First, biochemical and structural studies using techniques such as cryo-electron microscopy and single-molecule imaging could reveal how MCMs interact with damaged DNA and DDR factors. Second, genome-wide analyses, such as ChIP-seq and RNA-seq, can help identify global patterns of MCM binding and activity under different types of DNA damage. Third, the use of innovative model systems, including CRISPR-based gene editing and live-cell imaging, could provide insights into the dynamic regulation of MCMs in response to DNA damage and transcriptional stress. These approaches, combined with computational modeling, will enable a more comprehensive understanding of the complex roles of MCMs in DDR and their implications for genomic stability and human disease.

## 8. Conclusions and Perspective

**Figure 2:**
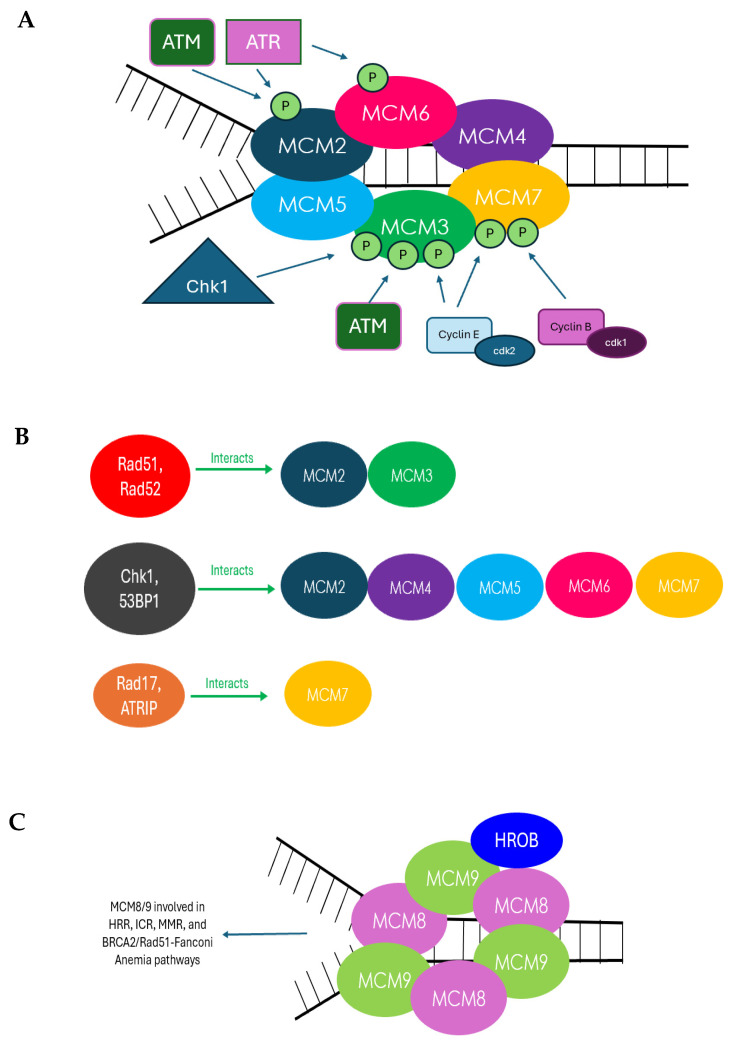

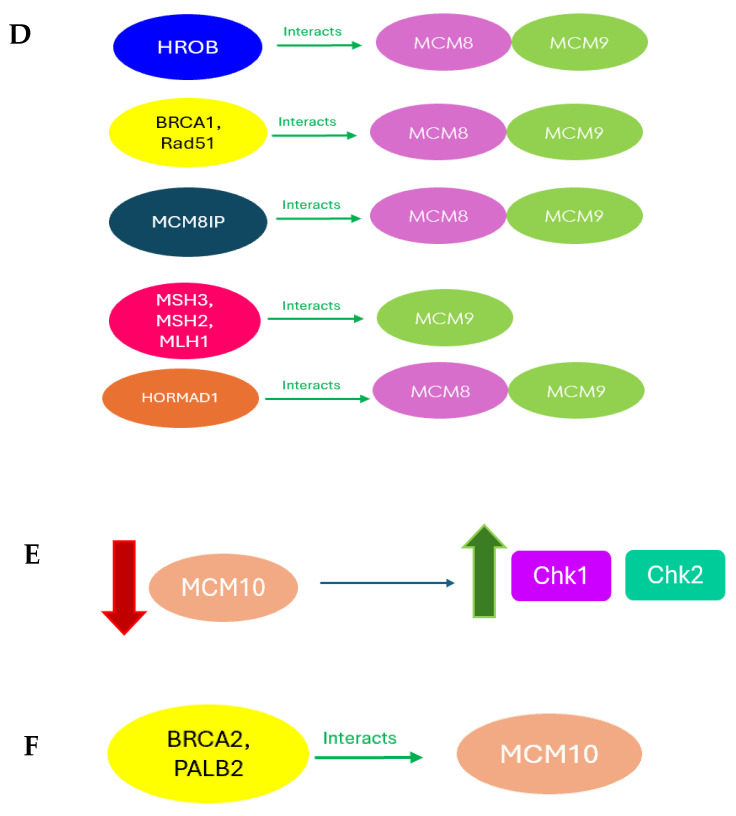
Summarizing the relationships between MCMs and DDR. (**A**) With respect to MCM2–7, known effects on MCM by the ATM/ATR pathway: ATM and ATR phosphorylate MCM2 on Serine 92, ATR phosphorylates MCM2 on Serine 108, cyclinE/Cdk2 phosphorylate MCM3 Threonine 722, Chk1 phosphorylates MCM3 on Serine 205, ATR phosphorylates MCM6 on Serine 13, Rad17 interacts with MCM7, MCM7 is phosphorylated on Serine 121 by cyclinE/Cdk2 and cyclinB/Cdk1. (**B**) Protein interactions between MCM2–7 and DDR proteins: MCM2 and MCM3 interact with Rad51 and Rad52MCM2/3 and /Rad51 promotes non-HRR repair, ATRIP interacts with MCM7, MCM3 interacts with Rad51 and Rad52; MCM2 interacts with Rad52, 53BP1 interacts with MCM 2/3/5/6. (**C**) MCM8/9 functions within DDR: MCM8 and MCM9 form a complex and are involved in HRR, MCM8 and MCM9 are involved in interstrand crosslink repair (ICL) repair and functions downstream of BRCA2/Rad51-Fanconi anemia pathways, and HROB recruits MCM8/9 to DNA damage sites. (**D**) Protein interactions between MCM8/9 and DDR proteins: MCM8IP interacts with MCM8/9, MCM8/9 interacts and directs BRCA1 and Rad51 to protect forks from excessive degradation, MCM9 interacts with mismatched repair (MMR) initiator proteins (MSH3, MSH2, MLH1), and HORMAD1 interacts with MCM8/MCM9. (**E**) MCM10 knockdown causes an increase in Chk1 and Chk2 expression, DNA breaks. (**F**) Known protein interactions between MCM10 and DDR proteins: MCM10 interacts with BRCA2 and PALB2.

In conclusion, the MCM family of proteins (MCM2–10) has demonstrated significant associations with DDR (Figure 2), as revealed through the series of studies, presented in this review, examining their roles in the cell cycle checkpoint activation and DNA repair pathways. While this review has sought to highlight all of the existing literature on the MCM-DDR connection, much remains to be discovered about this intricate relationship (Table 1). Further unraveling of these complexities could provide critical insights into how this connection can be leveraged to enhance our ability to interpret and treat diseases such as cancer. As mentioned, the overexpression of MCMs has been linked to tumor progression, positioning them as valuable biomarkers in oncology: MCM2–7 proteins show potential as sensitive diagnostic markers in hepatocellular carcinoma (HCC) [103], while MCM2 is implicated in gastric cardiac cancer and salivary gland tumors [104], MCM5 in esophageal cancer [105], MCM7 in meningiomas [106], and MCM10 in breast cancer [107]. These findings highlight the promise of MCM proteins in advancing cancer diagnosis and therapy.

As research into MCMs progresses, their potential as therapeutic targets continue to expand. Beyond their diagnostic value, the involvement of MCMs in DNA replication and repair pathways makes them attractive candidates for targeted cancer therapies. Inhibitors designed to disrupt MCM function could selectively impede the replication of rapidly dividing tumor cells while sparing normal cells, which rely on a more controlled replication process. Additionally, the modulation of post-translational modifications such as acetylation and deacetylation could provide novel strategies for regulating MCM activity in cancer cells, enhancing the efficacy of existing treatments. By bridging gaps in our understanding of MCM-DDR interactions, future studies may unlock innovative approaches to combating cancer and improving patient outcomes.

## Figures and Tables

**Table 1:** Summary of the relationship between MCMs and DDR.

MCMs	Notably Effects in DDR	Model	Reference
MCM2	MCM2 mutant was sensitive to MMS and caffeine. MCM2 regulates DNA replication in response to DNA damage	*Saccharomyces cerevisiae*	[57]
MCM2	DNA damage requires MCM2DENQ mutants to progress through G2/M	*Saccharomyces cerevisiae*	[59]
MCM2	DDK phosphorylates MCM2 to respond to replicative stress but not to induce checkpoint	*Saccharomyces pombe*	[75]
MCM2	ATM and ATR phosphorylate MCM2 on Serine 92	*Xenopus laevis*	[55]
MCM2	ATR phosphorylates MCM2 on Serine 108	HeLa and human dermal fibroblasts	[56]
MCM2	ATR phosphorylates MCM2 in response to pyrrole–imidazole polyamides	LNCaP, LNAR and DU145	[48]
MCM2	Cdc7/Dbf4 mediates phosphorylation on Serine 108 and Serine 40 of human MCM2 in the absence of DNA damage	A549 and HCT116	[49]
MCM3	CyclinE/Cdk2 phosphorylate MCM3 Threonine 722	HEK293-T and HeLa	[54]
MCM3	Chk1 phosphorylates MCM3 on Serine 205	HEK293-T, HeLa, U2OS, A549	[53]
MCM4	MCM4 interacts with Cds1 in response to HU MCM4 interacts with overexpressed Rad22 in response to HU MCM4 interacts with Rhp51 in response to HU	*Saccharomyces pombe*	[15]
MCM4	Early treatment of Widdrol leads to MCM4 downregulation only	HT-29 and SC-1	[66]
MCM4	Double mutant MCM4 and ATM led to decreased tumor latency and increased tumor susceptibility	Murine models	[62]
MCM4	N-terminal serine/threonine domain (NSD) on MCM4 mutants have elevated levels of Rad53 and increased γH2AX Deletion of NSD leads to weakened checkpoint response	*Saccharomyces cerevisiae*	[61]
MCM6	Mrc1 interacts with MCM6 and acts as checkpoint sensor for methanesulfonate (MMS)-induced DNA damage	*Saccharomyces cerevisiae*	[63]
MCM6	ATR phosphorylates MCM6 on Serine 13	U2OS	[65]
MCM7	Rad17 interacts with MCM7	U2OS and A549	[68]
MCM7	MCM7 is phosphorylated on Serine 121 by cyclin E/Cdk2 and cyclin B/Cdk1 MCM7 overexpression leads to S phase block Phosphorylation of MCM7 is necessary for proper mitotic exit	HEK293-T, HeLa and HCT116	[69]
MCM2 MCM3	MCM2 and MCM3 interact with Rad51 and Rad52. MCM2/3 and /Rad51 promotes non-HR repair	*Saccharomyces cerevisiae*	[47]
MCM2 MCM3 MCM7	ATR phosphorylates MCM2 on Serine 108 in response to ionizing radiation (IR), UV and Hydroxyurea (HU) ATM phosphorylates MCM3 on Serine 535 in response to IR ATRIP interacts with MCM7	HEK293, U2OS, HeLa	[30]
MCM2 MCM3	MCM3 siRNA led to G2 block and Chk1 and Chk2 activation MCM2 siRNA led to the activation of Chk2	HeLa	[19]
MCM2 MCM3	MCM2 and MCM3 knockdown causes reduction in Chk1 and Chk2 signaling in response to etoposide MCM2 and MCM3 knockdown causes reduction in HRR and NHEJ	HEK293 and U2OS	[31]
MCM2 MCM3	MCM3 interacts with Rad51 and Rad52 MCM2 interacts with Rad52	HeLa	[74]
MCM2 MCM5	MCM2 interacts with ASF1 in response to etoposide	U2OS	[32]
MCM2/3/5/6	53BP1 interacts with MCM 2/3/5/6. Knockdown of MCM2/6 reduces 53BP1 interaction with chromatin and reduces 53BP1 foci formation	HepG2	[72]
MCM2/3/MCM5/6	MDC1 interacts with MCM2/3/5/6. MDC1 interacts with MCM2/6 on chromatin	TE-1	[70]
MCM8 MCM9	Null MCM8 mice have blocked double-strand break repair. MCM8 and MCM9 form a complex and control HR	Murine modes and MEF	[77]
MCM8 MCM9	MCM 8 and MCM9 form a complex and are involved in HR MCM8 and MCM9 are involved in ICL repair and functions downstream of BRCA2/Rad51-Fanconi anemia pathways MCM8 and MCM9 are resistant to DNA interstrand crosslinks (ICL)	Chicken DT40	[78]
MCM8 MCM9	MCM8/9 are necessary for MRN localization to HR sites MCM8/9 are necessary for ssDNA generation for HR	U2OS, HeLa and HEK293-T	[79]
MCM8 MCM9	HROB recruits MCM8/9 to DNA damage sites	RPE1-hTERT, HCT116, HEK293-T, murine model	[80]
MCM8 MCM9	MCM8IP interacts with MCM8/9 MCM8IP stimulates the helicase activity of MCM8/9	HEK293T, HEK293T TREx, U2OS	[81]
MCM8 MCM9	MCM8/9 directs BRCA1 and Rad51 to protect forks from excessive degradation	HEK293	[85]
MCM8 MCM9	NLS motif is required for MCM8 and MCM9 nuclear localization BRC motif found in MCM9 directly interacts with and recruits Rad51 in response to mitomycin C DNA damage	HEK293-T and U2OS	[76]
MCM8 MCM9	Activated by HROB, the structure of MCM8/9 permits its unwinding ability	HeLa and Chicken DT40	[86]
MCM8 MCM9	HORMAD1 interacts with MCM8/MCM9 HORMAD1 disrupts MMR through its interaction with MCM8/9	HEK293-T and U2OS	[84]
MCM9	MCM9 interacts with mismatched repair (MMR) initiator proteins MCM9 is necessary for MMR and has helicase activity MCM9 helicase activity is necessary for MMR	HeLa	[83]
MCM10	Knockdown MCM10 causes an increase in Chk1 and Chk2 expression Prolonged knockdown of MCM10 causes DNA breaks	HeLa, S7, and YZ5	[87]
MCM10	MCM10 and DNA polymerase alpha interact Knockdown of MCM10 leads to Chk2 activation	HeLa	[39]
MCM10	MCM10 is downregulated in response to UV MCM10 overexpression helps cells recover from UV	U2OS and HeLa	[88]
MCM10	MCM10 interacts with BRCA2 and PALB2 MCM10-BRCA2 prevents fork progression in response to IR or bleomycin	CHO, VC8 and HEK293-T	[89]

## Data Availability

No new data were generated in this manuscript.
